# Supplementary material for: Sexual dimorphism in trait variability and its eco-evolutionary and statistical implications
Source: eLife. 2020 Nov 17;9:e63170. doi: 10.7554/eLife.63170 (PMC7704105; doi:10.7554/eLife.63170)
Supplement: Supplementary file 1. — Table 1. Summary of the available numbers of male and female mice from each strain and originating institution Table 2. Trait categories (parameter_group) and the number of correlated traits within these categories. Traits were meta-analysed using robumeta Table 3. We use this corrected (for correlated traits) results table, which contains each of the meta-analytic means for all effect sizes of interest, for further analyses. We further use this table as part of the Shiny App, which is able to provide the percentage differences between males and females for mean, variance and coefficient of variance (continued below) (Table 4). Summary of overall meta-analyses on the functional trait group level (GroupingTerm). Results for lnCVR, lnVR and lnRR and their respective upper and lower 95 percent CI’s, standard error and I2 values are provided. Values truncated at five decimal places for readability. Table 5. Provides an overview of meta-analysis results performed on traits that were significantly biased towards either sex. This table summarizes findings for both sexes and the respective functional trait groups. Values truncated at five decimal places for readability. Table 6. Summarizes our findings on heterogeneity due to institutions and mouse strains. These results are based on meta-analyses on sigma2 and errors for mouse strains and centres (Institutions), following the identical workflow from above. Values truncated at five decimal places for readability. [file elife-63170-supp1.docx]

Supplementary Tables to

**Sexual dimorphism in trait variability and its eco- evolutionary and statistical implications**

**Zajitschek, S.R.K.1,2 *, Zajitschek, F.1, Bonduriansky, R. 1, Brooks, R.C. 1, Cornwell, W.1, Falster, D.S.1, Lagisz, M. 1, Mason, J.3, Senior, A. M. 4, Noble, D. A. W.1,5 #, S. Nakagawa1 * #**

1 Evolution & Ecology Research Center, School of Biological, Earth, and Environmental Sciences, University of New South Wales, Sydney, 2052, NSW, Australia.

2 Liverpool John Moores University, School of Biological and Environmental Sciences, James Parsons Building, 3 Byrom Way, Liverpool L3 3 AF, UK.

3 European Bioinformatics Institute (EMBL-EBI), European Molecular Biology Laboratory, Wellcome Trust Genome Campus, Hinxton, Cambridge CB10 1SD, UK.

4 University of Sydney, Charles Perkins Centre, School of Life and Environmental Sciences, School of Mathematics and Statistics, Sydney, 2006, NSW, Australia.

5 Research School of Biology, Australian National University, Canberra, 2601, ACT, Australia.

# equal contributions as senior authors.
*corresponding authors:

S.Z. (susi.zajitschek@gmail.com) and S.N. (s.nakagawa@unsw.edu.au)

Table 1: Summary of the available numbers of male and female mice from each strain and originating institution

| production_center | strain_name | sex | n |
| --- | --- | --- | --- |
| BCM | C57BL/6N | female | 653 |
| BCM | C57BL/6N | male | 639 |
| BCM | C57BL/6N;C57BL/6NTac | female | 47 |
| BCM | C57BL/6N;C57BL/6NTac | male | 52 |
| BCM | C57BL/6NCrl | female | 4 |
| BCM | C57BL/6NCrl | male | 2 |
| BCM | C57BL/6NJ | female | 6 |
| BCM | C57BL/6NJ | male | 6 |
| BCM | C57BL/6NTac | female | 1 |
| BCM | C57BL/6NTac | male | 5 |
| HMGU | C57BL/6NCrl | female | 313 |
| HMGU | C57BL/6NCrl | male | 311 |
| HMGU | C57BL/6NTac | female | 1045 |
| HMGU | C57BL/6NTac | male | 1062 |
| ICS | C57BL/6N | female | 1025 |
| ICS | C57BL/6N | male | 1050 |
| JAX | C57BL/6NJ | female | 2025 |
| JAX | C57BL/6NJ | male | 2022 |
| KMPC | C57BL/6N;C57BL/6NTac | female | 271 |
| KMPC | C57BL/6N;C57BL/6NTac | male | 266 |
| MARC | C57BL/6N | female | 936 |
| MARC | C57BL/6N | male | 926 |
| MRC Harwell | C57BL/6NTac | female | 2639 |
| MRC Harwell | C57BL/6NTac | male | 2661 |
| MRC Harwell | C57BL/6NTac | no_data | 3 |
| RBRC | C57BL/6NJcl | female | 222 |
| RBRC | C57BL/6NJcl | male | 222 |
| RBRC | C57BL/6NTac | female | 526 |
| RBRC | C57BL/6NTac | male | 523 |
| TCP | C57BL/6NCrl | female | 552 |
| TCP | C57BL/6NCrl | male | 524 |
| TCP | C57BL6/NCrl | female | 2 |
| TCP | C57BL6/NCrl | male | 2 |
| UC Davis | C57BL/6N | male | 1 |
| UC Davis | C57BL/6NCrl | female | 1155 |
| UC Davis | C57BL/6NCrl | male | 1158 |
| WTSI | B6Brd;B6Dnk;B6N-Tyr<c-Brd> | female | 97 |
| WTSI | B6Brd;B6Dnk;B6N-Tyr<c-Brd> | male | 87 |
| WTSI | C57BL/6NTac/USA | male | 3 |
| WTSI | C57BL/6N | female | 1951 |
| WTSI | C57BL/6N | male | 2008 |
| WTSI | C57BL/6N;C57BL/6NTac | female | 41 |
| WTSI | C57BL/6N;C57BL/6NTac | male | 7 |
| WTSI | C57BL/6NCrl | male | 13 |
| WTSI | C57BL/6NTac | female | 49 |
| WTSI | C57BL/6NTac | male | 34 |

Table 2: Trait categories (parameter_group) and the number of correlated traits within these categories. Traits were meta-analysed using robumeta

| parameter_group | n |
| --- | --- |
| 12khz-evoked abr threshold | 1 |
| 18khz-evoked abr threshold | 1 |
| 24khz-evoked abr threshold | 1 |
| 30khz-evoked abr threshold | 1 |
| 6khz-evoked abr threshold | 1 |
| alanine aminotransferase | 1 |
| albumin | 1 |
| alkaline phosphatase | 1 |
| alpha-amylase | 1 |
| area under glucose response curve | 1 |
| aspartate aminotransferase | 1 |
| B cells | 4 |
| basophil cell count | 1 |
| basophil differential count | 1 |
| bmc/body weight | 1 |
| body length | 1 |
| body temp | 1 |
| body weight | 1 |
| body weight after experiment | 1 |
| body weight before experiment | 1 |
| bone area | 1 |
| bone mineral content (excluding skull) | 1 |
| bone mineral density (excluding skull) | 1 |
| calcium | 1 |
| cardiac output | 1 |
| cd4 nkt | 6 |
| cd4 t | 7 |
| cd8 nkt | 6 |
| cd8 t | 7 |
| cdcs | 2 |
| center average speed | 1 |
| center distance travelled | 1 |
| center permanence time | 1 |
| center resting time | 1 |
| chloride | 1 |
| click-evoked abr threshold | 1 |
| creatine kinase | 1 |
| creatinine | 1 |
| cv | 1 |
| distance travelled - total | 1 |
| dn nkt | 6 |
| dn t | 7 |
| ejection fraction | 1 |
| end-diastolic diameter | 1 |
| end-systolic diameter | 1 |
| eosinophils | 3 |
| fasted blood glucose concentration | 1 |
| fat mass | 1 |
| fat/body weight | 1 |
| follicular b cells | 2 |
| forelimb and hindlimb grip strength measurement mean | 1 |
| forelimb grip strength measurement mean | 1 |
| fractional shortening | 1 |
| free fatty acids | 1 |
| fructosamine | 1 |
| glucose | 1 |
| hdl-cholesterol | 1 |
| heart weight | 1 |
| heart weight normalised against body weight | 1 |
| hematocrit | 1 |
| hemoglobin | 1 |
| hr | 1 |
| hrv | 1 |
| initial response to glucose challenge | 1 |
| insulin | 1 |
| iron | 1 |
| lactate dehydrogenase | 1 |
| latency to center entry | 1 |
| ldl-cholesterol | 1 |
| lean mass | 1 |
| lean/body weight | 1 |
| left anterior chamber depth | 1 |
| left corneal thickness | 1 |
| left inner nuclear layer | 1 |
| left outer nuclear layer | 1 |
| left posterior chamber depth | 1 |
| left total retinal thickness | 1 |
| locomotor activity | 1 |
| luc | 2 |
| lvawd | 1 |
| lvaws | 1 |
| lvidd | 1 |
| lvids | 1 |
| lvpwd | 1 |
| lvpws | 1 |
| lymphocytes | 2 |
| magnesium | 1 |
| mean cell hemoglobin concentration | 1 |
| mean cell volume | 1 |
| mean corpuscular hemoglobin | 1 |
| mean platelet volume | 1 |
| mean r amplitude | 1 |
| mean sr amplitude | 1 |
| monocytes | 3 |
| neutrophils | 3 |
| nk cells | 6 |
| nkt cells | 4 |
| number of center entries | 1 |
| number of rears - total | 1 |
| others | 1 |
| pdcs | 1 |
| percentage center time | 1 |
| percentage of live gated events | 2 |
| periphery average speed | 1 |
| periphery distance travelled | 1 |
| periphery permanence time | 1 |
| periphery resting time | 1 |
| phosphorus | 1 |
| platelet count | 1 |
| pnn5(6>ms) | 1 |
| potassium | 1 |
| pq | 1 |
| pr | 1 |
| pre-pulse inhibition | 5 |
| qrs | 1 |
| qtc | 1 |
| qtc dispersion | 1 |
| red blood cell count | 1 |
| red blood cell distribution width | 1 |
| respiration rate | 1 |
| respiratory exchange ratio | 1 |
| response amplitude | 10 |
| right anterior chamber depth | 1 |
| right corneal thickness | 1 |
| right inner nuclear layer | 1 |
| right outer nuclear layer | 1 |
| right posterior chamber depth | 1 |
| right total retinal thickness | 1 |
| rmssd | 1 |
| rp macrophage (cd19- cd11c-) | 1 |
| rr | 1 |
| sodium | 1 |
| spleen weight | 1 |
| st | 1 |
| stroke volume | 1 |
| t cells | 3 |
| tibia length | 1 |
| total bilirubin | 1 |
| total cholesterol | 1 |
| total food intake | 1 |
| total protein | 1 |
| total water intake | 1 |
| triglycerides | 1 |
| urea (blood urea nitrogen - bun) | 1 |
| uric acid | 1 |
| white blood cell count | 1 |
| whole arena average speed | 1 |
| whole arena resting time | 1 |

Table 3: We use this corrected (for correlated traits) results table, which contains each of the meta-analytic means for all effect sizes of interest, for further analyses. We further use this table as part of the Shiny App, which is able to provide the percentage differences between males and females for mean, variance and coefficient of variance. (continued below)

| parameter_  group | counts | procedure | Grouping  Term | lnCVR | lnCVR_lwr | lnCVR_uppr | lnCVR_se | lnVR | lnVR_lwe | lnVR_uppe | lnVR_se | lnRR | lnRR_lwr | lnRR_uppr | lnRR_se |
| --- | --- | --- | --- | --- | --- | --- | --- | --- | --- | --- | --- | --- | --- | --- | --- |
| pre-pulse inhibition | 5 | Acoustic Startle and Pre-pulse Inhibition (PPI) | Behaviour | 0.02329626 | -0.0802563 | 0.12684879 | 0.03705066 | 0.00910276 | -0.036464 | 0.05466951 | 0.01434306 | -0.0052156 | -0.0427126 | 0.03228149 | 0.01280924 |
| B cells | 4 | Immunophenotyping | Immunology | -0.0938959 | -0.250002 | 0.06221027 | 0.04269721 | -0.0995337 | -0.2068001 | 0.00773283 | 0.02501316 | -0.0026281 | -0.129823 | 0.12456678 | 0.03930176 |
| cd4 nkt | 6 | Immunophenotyping | Immunology | -0.0287688 | -0.0566987 | -8.39E-04 | 0.01016335 | -0.2018746 | -0.3102294 | -0.0935198 | 0.03311612 | -0.234445 | -0.4005266 | -0.0683635 | 0.06335011 |
| cd4 t | 7 | Immunophenotyping | Immunology | -0.1507387 | -0.2427976 | -0.0586798 | 0.036069 | -0.1699213 | -0.262945 | -0.0768975 | 0.03483244 | -0.0031242 | -0.0411564 | 0.03490806 | 0.01489891 |
| cd8 nkt | 6 | Immunophenotyping | Immunology | -0.0424402 | -0.0782046 | -0.0066759 | 0.01192233 | -0.0300442 | -0.1823594 | 0.12227104 | 0.05337653 | 0.00353724 | -0.0573749 | 0.06444936 | 0.0205272 |
| cd8 t | 7 | Immunophenotyping | Immunology | -0.1223681 | -0.2179976 | -0.0267387 | 0.03587268 | -0.1581698 | -0.2342579 | -0.0820816 | 0.02702292 | -0.0415806 | -0.0510391 | -0.0321221 | 0.00231186 |
| cdcs | 2 | Immunophenotyping | Immunology | -0.0362947 | -0.3588637 | 0.28627421 | 0.02538673 | 0.10802477 | -0.0565718 | 0.27262133 | 0.01295403 | 0.16425405 | -0.170152 | 0.49866012 | 0.02631833 |
| dn nkt | 6 | Immunophenotyping | Immunology | -0.0619371 | -0.135938 | 0.01206372 | 0.02577458 | -0.1572129 | -0.2814342 | -0.0329915 | 0.04471627 | -0.1727105 | -0.2906356 | -0.0547854 | 0.04410337 |
| dn t | 7 | Immunophenotyping | Immunology | -0.0796127 | -0.1844481 | 0.02522265 | 0.0420063 | -0.2421038 | -0.3431678 | -0.1410397 | 0.04063142 | -0.2298147 | -0.2519708 | -0.2076586 | 0.00723731 |
| eosinophils | 3 | Hematology | Hematology | -0.0662225 | -0.2806631 | 0.14821812 | 0.03258591 | -0.0154112 | -0.4051652 | 0.37434268 | 0.08653658 | -0.0042422 | -0.2409206 | 0.23243619 | 0.05080934 |
| follicular b cells | 2 | Immunophenotyping | Immunology | -0.1160077 | -0.7256692 | 0.49365384 | 0.0479814 | -0.1050194 | -0.6946364 | 0.48459772 | 0.04640387 | 0.00524271 | -0.1872381 | 0.19772355 | 0.01514857 |
| luc | 2 | Hematology | Hematology | 0.01804357 | -0.2038464 | 0.23993356 | 0.01746312 | 0.2657035 | -1.2251358 | 1.75654278 | 0.1173316 | 0.22154965 | -1.4136389 | 1.85673819 | 0.12869213 |
| lymphocytes | 2 | Hematology | Hematology | 0.08052298 | -2.2618128 | 2.42285879 | 0.18434583 | 0.1550159 | -1.0892706 | 1.39930242 | 0.09792747 | 0.06021444 | -1.0131287 | 1.1335576 | 0.08447394 |
| monocytes | 3 | Hematology | Hematology | -0.0214677 | -0.2033706 | 0.16043516 | 0.04206047 | 0.07848762 | -0.1811005 | 0.33807574 | 0.0585593 | 0.10251934 | -0.1483375 | 0.35337622 | 0.05714382 |
| neutrophils | 3 | Hematology | Hematology | 0.25874459 | 0.01308031 | 0.50440886 | 0.05575156 | 0.37998053 | -0.2060446 | 0.96600568 | 0.13179801 | 0.13193722 | -0.2669324 | 0.53080682 | 0.09243361 |
| nk cells | 6 | Immunophenotyping | Immunology | -0.0414772 | -0.0960406 | 0.01308625 | 0.02004114 | 0.01565331 | -0.0703789 | 0.10168557 | 0.03154872 | 0.04717574 | -0.0162213 | 0.11057281 | 0.02318305 |
| nkt cells | 4 | Immunophenotyping | Immunology | 0.00337567 | -0.106989 | 0.11374038 | 0.02946609 | -0.2458705 | -0.4452333 | -0.0465077 | 0.04267377 | -0.1823355 | -0.3233946 | -0.0412763 | 0.03145804 |
| percentage of live gated events | 2 | Immunophenotyping | Immunology | -0.0934933 | -0.303734 | 0.11674731 | 0.0165463 | -0.0412606 | -0.1414443 | 0.05892307 | 0.00788463 | 0.05009409 | 0.00811913 | 0.09206905 | 0.0033035 |
| response amplitude | 10 | Acoustic Startle and Pre-pulse Inhibition (PPI) | Behaviour | 0.03331468 | -0.0127585 | 0.07938788 | 0.02029469 | 0.25492738 | 0.19697866 | 0.3128761 | 0.02550027 | 0.20160617 | 0.11081365 | 0.29239869 | 0.04011639 |
| t cells | 3 | Immunophenotyping | Immunology | -0.1338701 | -0.2750284 | 0.00728828 | 0.03265942 | -0.1240786 | -0.4120104 | 0.16385312 | 0.06686111 | -5.75E-04 | -0.1663201 | 0.16517023 | 0.0374233 |
| 12khz-evoked abr threshold | 1 | Auditory Brain Stem Response | Hearing | 0.05386546 | -0.005683 | 0.11341389 | 0.03038241 | 0.08696495 | 0.00658023 | 0.16734967 | 0.04101337 | 0.00248509 | -0.0214504 | 0.02642053 | 0.01221219 |
| 18khz-evoked abr threshold | 1 | Auditory Brain Stem Response | Hearing | 0.02382414 | -0.0331809 | 0.08082923 | 0.02908476 | 0.02502657 | -0.048845 | 0.09889817 | 0.03769029 | -0.0200763 | -0.0431508 | 0.00299819 | 0.01177291 |
| 24khz-evoked abr threshold | 1 | Auditory Brain Stem Response | Hearing | 0.05181273 | -0.0148242 | 0.1184497 | 0.03399908 | -0.089151 | -0.3321998 | 0.15389771 | 0.12400674 | -0.0224536 | -0.0444163 | -4.91E-04 | 0.01120565 |
| 30khz-evoked abr threshold | 1 | Auditory Brain Stem Response | Hearing | 0.01709326 | -0.0533187 | 0.08750527 | 0.03592515 | -0.0344797 | -0.1017901 | 0.03283061 | 0.03434265 | -0.0497874 | -0.0748197 | -0.024755 | 0.01277184 |
| 6khz-evoked abr threshold | 1 | Auditory Brain Stem Response | Hearing | -0.0077678 | -0.0418582 | 0.02632256 | 0.01739337 | 0.01416819 | -0.0189973 | 0.0473337 | 0.01692149 | 0.01840433 | 0.00568972 | 0.03111894 | 0.00648716 |
| alanine aminotransferase | 1 | Clinical Chemistry | Physiology | -0.0684217 | -0.189502 | 0.05265861 | 0.0617768 | 0.05851792 | -0.1322507 | 0.2492866 | 0.09733274 | 0.1069442 | 0.03199344 | 0.18189496 | 0.03824089 |
| albumin | 1 | Clinical Chemistry | Physiology | 0.113308 | 0.04514753 | 0.18146846 | 0.03477638 | 0.05599948 | -0.0080678 | 0.12006678 | 0.032688 | -0.056784 | -0.0732083 | -0.0403597 | 0.0083799 |
| alkaline phosphatase | 1 | Clinical Chemistry | Physiology | 0.10436489 | 0.04515852 | 0.16357127 | 0.03020789 | -0.3112471 | -0.3980164 | -0.2244778 | 0.04427086 | -0.4216032 | -0.4694832 | -0.3737231 | 0.02442903 |
| alpha-amylase | 1 | Clinical Chemistry | Physiology | 0.03834067 | -0.0423419 | 0.1190232 | 0.04116531 | 0.27955657 | 0.16157766 | 0.39753547 | 0.06019443 | 0.22469866 | 0.17931513 | 0.27008219 | 0.02315529 |
| area under glucose response curve | 1 | Intraperitoneal glucose tolerance test (IPGTT) | Metabolism | -0.1531723 | -0.2210551 | -0.0852895 | 0.03463471 | 0.27483964 | 0.1950895 | 0.35458977 | 0.04068959 | 0.43577382 | 0.36558816 | 0.50595949 | 0.03580967 |
| aspartate aminotransferase | 1 | Clinical Chemistry | Physiology | 0.01191646 | -0.1228287 | 0.14666166 | 0.06874882 | -0.0566968 | -0.2457779 | 0.13238433 | 0.09647173 | -0.0585577 | -0.1331777 | 0.01606241 | 0.03807216 |
| basophil cell count | 1 | Hematology | Hematology | -0.0917931 | -0.2022487 | 0.01866245 | 0.05635592 | 0.20312654 | -0.0131549 | 0.41940795 | 0.11034968 | 0.26757721 | 0.06430284 | 0.47085158 | 0.10371332 |
| basophil differential count | 1 | Hematology | Hematology | -0.0934739 | -0.1787512 | -0.0081966 | 0.04350962 | -0.0639511 | -0.2828066 | 0.15490445 | 0.11166303 | -0.0156339 | -0.110231 | 0.07896328 | 0.04826475 |
| bmc/body weight | 1 | Body Composition (DEXA lean/fat) | Morphology | 0.13149984 | 0.03298458 | 0.23001511 | 0.05026381 | -0.0448684 | -0.1340146 | 0.04427775 | 0.04548358 | -0.1722378 | -0.220703 | -0.1237726 | 0.0247276 |
| body length | 1 | Body Composition (DEXA lean/fat) | Morphology | -0.0347988 | -0.0824528 | 0.01285522 | 0.02431371 | -0.0059677 | -0.0526221 | 0.04068664 | 0.02380368 | 0.02827217 | 0.02332538 | 0.03321895 | 0.00252391 |
| body temp | 1 | Echo | Heart | -0.0325368 | -0.1066429 | 0.04156933 | 0.03780994 | -0.0303742 | -0.1044537 | 0.04370539 | 0.03779638 | 0.00185319 | -5.00E-04 | 0.00420662 | 0.00120075 |
| body weight | 1 | Body Weight | Morphology | 0.02456752 | -0.0420402 | 0.09117522 | 0.03398414 | 0.23357933 | 0.16949794 | 0.29766072 | 0.03269519 | 0.209677 | 0.19387273 | 0.22548127 | 0.00806355 |
| body weight after experiment | 1 | Indirect Calorimetry | Metabolism | 0.08537078 | 0.02996646 | 0.1407751 | 0.02826803 | 0.28493705 | 0.23288753 | 0.33698657 | 0.02655637 | 0.20309733 | 0.1864076 | 0.21978706 | 0.00851533 |
| body weight before experiment | 1 | Indirect Calorimetry | Metabolism | 0.10535114 | 0.04124611 | 0.16945618 | 0.03270725 | 0.30389983 | 0.24354284 | 0.36425682 | 0.03079495 | 0.20086377 | 0.18163616 | 0.22009138 | 0.00981019 |
| bone area | 1 | Body Composition (DEXA lean/fat) | Morphology | 0.09815869 | 0.02728245 | 0.16903494 | 0.03616201 | 0.12865455 | 0.05336593 | 0.20394318 | 0.03841327 | 0.03152409 | 3.81E-04 | 0.06266758 | 0.01588983 |
| bone mineral content (excluding skull) | 1 | Body Composition (DEXA lean/fat) | Morphology | 0.17092299 | 0.06256422 | 0.27928176 | 0.0552861 | 0.20913716 | 0.10155997 | 0.31671434 | 0.05488733 | 0.03725371 | -0.0130828 | 0.08759021 | 0.02568236 |
| bone mineral density (excluding skull) | 1 | Body Composition (DEXA lean/fat) | Morphology | 0.05426375 | -0.0881612 | 0.19668869 | 0.07266712 | 0.04928301 | -0.1087868 | 0.20735284 | 0.08064935 | 0.00122862 | -0.0187942 | 0.02125141 | 0.0102159 |
| calcium | 1 | Clinical Chemistry | Physiology | 0.0097946 | -0.04646 | 0.06604915 | 0.02870183 | 0.01356828 | -0.04246 | 0.06959659 | 0.0285864 | 0.00365644 | -6.09E-05 | 0.00737374 | 0.00189662 |
| cardiac output | 1 | Echo | Heart | 0.01338156 | -0.0797535 | 0.10651665 | 0.04751878 | 0.10179908 | 0.02062872 | 0.18296944 | 0.04141421 | 0.09344391 | 0.0580233 | 0.12886452 | 0.01807207 |
| center average speed | 1 | Open Field | Behaviour | 0.01673 | -0.0404735 | 0.07393351 | 0.029186 | -0.0588515 | -0.1004209 | -0.017282 | 0.0212093 | -0.0724619 | -0.1149622 | -0.0299616 | 0.02168422 |
| center distance travelled | 1 | Open Field | Behaviour | -0.0162603 | -0.0733243 | 0.04080382 | 0.02911486 | -0.1060637 | -0.2023343 | -0.009793 | 0.0491186 | -0.0940204 | -0.1945774 | 0.0065366 | 0.05130554 |
| center permanence time | 1 | Open Field | Behaviour | -0.0253715 | -0.0826435 | 0.03190041 | 0.02922092 | -0.0255734 | -0.1014389 | 0.05029219 | 0.03870763 | -0.0035151 | -0.0902886 | 0.08325845 | 0.04427302 |
| center resting time | 1 | Open Field | Behaviour | 0.0244492 | -0.0737922 | 0.12269057 | 0.05012407 | -0.022869 | -0.1548339 | 0.10909595 | 0.06733029 | -0.0630751 | -0.2215457 | 0.09539555 | 0.08085384 |
| chloride | 1 | Clinical Chemistry | Physiology | 0.03215554 | -0.1270972 | 0.19140826 | 0.08125288 | 0.02414915 | -0.1438502 | 0.19214848 | 0.08571552 | -0.0127047 | -0.0177349 | -0.0076745 | 0.00256647 |
| click-evoked abr threshold | 1 | Auditory Brain Stem Response | Hearing | -0.052945 | -0.1534816 | 0.04759152 | 0.05129509 | -0.0561198 | -0.1827679 | 0.07052823 | 0.06461755 | -0.0154221 | -0.05772 | 0.02687573 | 0.02158093 |
| creatine kinase | 1 | Clinical Chemistry | Physiology | 0.02412316 | -0.1071457 | 0.155392 | 0.06697513 | -0.1318792 | -0.3968974 | 0.13313903 | 0.13521587 | -0.1344413 | -0.3838303 | 0.11494764 | 0.1272416 |
| creatinine | 1 | Clinical Chemistry | Physiology | 0.03523149 | -0.0229205 | 0.09338345 | 0.02966991 | 0.10663733 | -0.2200831 | 0.43335779 | 0.16669718 | -0.0844078 | -0.1320251 | -0.0367905 | 0.02429497 |
| cv | 1 | Electrocardiogram (ECG) | Heart | 0.18745439 | 0.07166312 | 0.30324567 | 0.05907827 | -0.0895722 | -0.2484833 | 0.06933882 | 0.08107855 | -0.2401301 | -0.3410322 | -0.139228 | 0.05148161 |
| distance travelled - total | 1 | Open Field | Behaviour | -0.0187819 | -0.0858957 | 0.04833185 | 0.03424234 | -0.1272582 | -0.1997426 | -0.0547738 | 0.03698252 | -0.1121373 | -0.1816322 | -0.0426424 | 0.03545723 |
| ejection fraction | 1 | Echo | Heart | -0.0300111 | -0.1345066 | 0.07448444 | 0.05331501 | -0.0525735 | -0.1483174 | 0.04317046 | 0.04884985 | -0.0284086 | -0.0492579 | -0.0075592 | 0.01063761 |
| end-diastolic diameter | 1 | Echo | Heart | 0.11209716 | 0.04314889 | 0.18104542 | 0.03517833 | 0.17439293 | 0.08752517 | 0.2612607 | 0.0443211 | 0.06009069 | 0.03549231 | 0.08468907 | 0.01255042 |
| end-systolic diameter | 1 | Echo | Heart | -0.0084176 | -0.0780811 | 0.06124591 | 0.03554326 | 0.06689658 | -0.0016692 | 0.1354624 | 0.0349832 | 0.0763195 | 0.04511358 | 0.10752543 | 0.01592168 |
| fasted blood glucose concentration | 1 | Intraperitoneal glucose tolerance test (IPGTT) | Metabolism | -0.0177245 | -0.1256855 | 0.09023658 | 0.05508318 | 0.07028239 | -0.0302439 | 0.17080868 | 0.05128986 | 0.086842 | 0.04930074 | 0.12438325 | 0.01915405 |
| fat mass | 1 | Body Composition (DEXA lean/fat) | Morphology | 0.04087989 | -0.0430149 | 0.12477464 | 0.04280423 | 0.37143133 | 0.26987898 | 0.47298367 | 0.05181337 | 0.32820804 | 0.26690318 | 0.38951289 | 0.03127856 |
| fat/body weight | 1 | Body Composition (DEXA lean/fat) | Morphology | 0.07773274 | -0.0119735 | 0.16743898 | 0.04576933 | 0.20207765 | 0.10835572 | 0.29579957 | 0.04781819 | 0.12352921 | 0.06386291 | 0.18319551 | 0.03044255 |
| forelimb and hindlimb grip strength measurement mean | 1 | Grip Strength | Morphology | 0.05781581 | 0.00399979 | 0.11163184 | 0.02745766 | 0.11459856 | 0.05305207 | 0.17614505 | 0.03140185 | 0.05418883 | 0.02948383 | 0.07889383 | 0.01260482 |
| forelimb grip strength measurement mean | 1 | Grip Strength | Morphology | 0.02650507 | -0.018724 | 0.07173414 | 0.02307648 | 0.09950765 | 0.05397397 | 0.14504132 | 0.02323189 | 0.06970607 | 0.04386251 | 0.09554963 | 0.01318573 |
| fractional shortening | 1 | Echo | Heart | -0.0148852 | -0.1161666 | 0.08639613 | 0.05167512 | -0.0575326 | -0.1558559 | 0.04079071 | 0.05016587 | -0.0413498 | -0.0567105 | -0.0259891 | 0.00783724 |
| free fatty acids | 1 | Clinical Chemistry | Physiology | 0.02815758 | -0.1002531 | 0.15656829 | 0.06551687 | 0.05541089 | -0.0736861 | 0.18450793 | 0.06586705 | 0.0193783 | -0.00937 | 0.04812664 | 0.01466779 |
| fructosamine | 1 | Clinical Chemistry | Physiology | -0.0397864 | -0.1198801 | 0.04030734 | 0.04086489 | -0.0678231 | -0.1513538 | 0.01570749 | 0.04261845 | -0.0283579 | -0.0692447 | 0.01252892 | 0.02086101 |
| glucose | 1 | Clinical Chemistry | Physiology | 0.06926007 | 0.01840253 | 0.12011761 | 0.0259482 | 0.12794733 | 0.04230008 | 0.21359458 | 0.04369838 | 0.06508874 | 0.02184962 | 0.10832785 | 0.02206118 |
| hdl-cholesterol | 1 | Clinical Chemistry | Physiology | -0.0650177 | -0.1255786 | -0.0044568 | 0.03089898 | 0.17243539 | 0.07010619 | 0.2747646 | 0.05220974 | 0.26069612 | 0.21804213 | 0.30335012 | 0.02176264 |
| heart weight | 1 | Heart Weight | Morphology | 0.17668315 | 0.06728427 | 0.28608204 | 0.05581678 | 0.3651806 | 0.21698396 | 0.51337724 | 0.07561192 | 0.17376154 | 0.14090374 | 0.20661935 | 0.01676449 |
| heart weight normalised against body weight | 1 | Heart Weight | Morphology | 0.07943035 | -0.0060591 | 0.16491981 | 0.04361787 | 0.03555735 | -0.0973272 | 0.16844189 | 0.06779948 | -0.0495578 | -0.0835809 | -0.0155346 | 0.01735906 |
| hematocrit | 1 | Hematology | Hematology | 0.05663564 | -0.0516862 | 0.16495748 | 0.05526726 | 0.0737071 | -0.0328632 | 0.18027744 | 0.05437362 | 0.01739665 | 0.0035179 | 0.03127541 | 0.00708113 |
| hemoglobin | 1 | Hematology | Hematology | 0.08669999 | 0.0269936 | 0.14640639 | 0.03046301 | 0.08673452 | 0.01940222 | 0.15406682 | 0.03435385 | 0.00519918 | -0.0080216 | 0.01841994 | 0.00674541 |
| hr | 1 | Electrocardiogram (ECG) | Heart | -0.063449 | -0.1734699 | 0.04657182 | 0.05613413 | -0.0140315 | -0.1488474 | 0.12078433 | 0.06878487 | 0.04066172 | -0.0139214 | 0.09524483 | 0.02784904 |
| hrv | 1 | Electrocardiogram (ECG) | Heart | 0.17225931 | 0.1094294 | 0.23508921 | 0.03205666 | -0.0813225 | -0.2125462 | 0.04990114 | 0.06695209 | -0.250499 | -0.3657436 | -0.1352545 | 0.05879931 |
| initial response to glucose challenge | 1 | Intraperitoneal glucose tolerance test (IPGTT) | Metabolism | -0.0968821 | -0.150378 | -0.0433861 | 0.02729435 | 0.04299714 | 0.01418065 | 0.07181364 | 0.01470256 | 0.11836258 | 0.08532424 | 0.15140092 | 0.01685661 |
| insulin | 1 | Insulin Blood Level | Metabolism | -0.0993292 | -0.3721975 | 0.17353908 | 0.13922107 | 0.17740025 | -0.1938091 | 0.54860959 | 0.189396 | 0.4445455 | 0.09444976 | 0.79464124 | 0.17862356 |
| iron | 1 | Clinical Chemistry | Physiology | -0.0974214 | -0.2141737 | 0.01933097 | 0.05956861 | -0.2534898 | -0.3963648 | -0.1106147 | 0.07289678 | -0.1527977 | -0.1930307 | -0.1125646 | 0.02052744 |
| lactate dehydrogenase | 1 | Clinical Chemistry | Physiology | 0.09412486 | -0.0214022 | 0.20965191 | 0.05894345 | 0.14092696 | -0.0620594 | 0.34391333 | 0.10356638 | 0.03188006 | -0.1412218 | 0.20498189 | 0.08831888 |
| latency to center entry | 1 | Open Field | Behaviour | 0.1254239 | 0.03301848 | 0.21782933 | 0.04714649 | 0.36412207 | 0.20560002 | 0.52264412 | 0.08088008 | 0.27345188 | 0.07393659 | 0.47296718 | 0.10179539 |
| ldl-cholesterol | 1 | Clinical Chemistry | Physiology | 0.42316441 | 0.15517757 | 0.69115125 | 0.13673049 | 0.26692834 | -0.0956833 | 0.62953996 | 0.18500933 | -0.1615499 | -0.6010478 | 0.27794804 | 0.22423775 |
| lean mass | 1 | Body Composition (DEXA lean/fat) | Morphology | 0.14357562 | 0.07593424 | 0.21121699 | 0.03451154 | 0.33824469 | 0.2664863 | 0.41000308 | 0.0366121 | 0.19289451 | 0.17524252 | 0.2105465 | 0.00900628 |
| lean/body weight | 1 | Body Composition (DEXA lean/fat) | Morphology | 0.19538328 | 0.09124799 | 0.29951858 | 0.05313123 | 0.18407857 | 0.08637641 | 0.28178072 | 0.04984895 | -0.0122785 | -0.0257504 | 0.0011934 | 0.00687355 |
| left anterior chamber depth | 1 | Eye Morphology | Eye | -0.1854856 | -0.4305058 | 0.05953466 | 0.12501263 | -0.1534983 | -0.4007283 | 0.09373164 | 0.12614007 | 0.03317463 | 0.0284172 | 0.03793206 | 0.00242731 |
| left corneal thickness | 1 | Eye Morphology | Eye | -0.1446634 | -0.233995 | -0.0553319 | 0.04557815 | -0.1352252 | -0.2234178 | -0.0470327 | 0.04499702 | 0.00752828 | -0.0057082 | 0.02076481 | 0.00675345 |
| left inner nuclear layer | 1 | Eye Morphology | Eye | 0.04804582 | -0.0360706 | 0.13216223 | 0.04291732 | 0.04872172 | -0.0347622 | 0.13220567 | 0.04259464 | 6.96E-04 | -0.0095012 | 0.0108923 | 0.00520251 |
| left outer nuclear layer | 1 | Eye Morphology | Eye | -0.0675012 | -0.1511666 | 0.01616412 | 0.04268718 | -0.0618025 | -0.1452865 | 0.02168141 | 0.04259464 | 0.00638113 | 0.00117015 | 0.01159211 | 0.00265871 |
| left posterior chamber depth | 1 | Eye Morphology | Eye | -0.2631046 | -0.4734756 | -0.0527336 | 0.10733412 | -0.268736 | -0.4790035 | -0.0584686 | 0.10728127 | -0.0026027 | -0.0146655 | 0.00946004 | 0.00615458 |
| left total retinal thickness | 1 | Eye Morphology | Eye | -0.197577 | -0.4386627 | 0.0435087 | 0.12300516 | -0.1932648 | -0.4269751 | 0.04044563 | 0.11924219 | 0.00279954 | -0.0034907 | 0.00908977 | 0.00320936 |
| locomotor activity | 1 | Combined SHIRPA and Dysmorphology | Behaviour | 0.09601056 | 0.02242142 | 0.1695997 | 0.03754617 | -0.0159064 | -0.0579694 | 0.02615656 | 0.0214611 | -0.1105803 | -0.1761043 | -0.0450562 | 0.03343127 |
| lvawd | 1 | Echo | Heart | 0.02289241 | -0.0247048 | 0.07048964 | 0.02428475 | 0.04540752 | -0.0013249 | 0.09213993 | 0.0238435 | 0.02466136 | 0.01140951 | 0.03791321 | 0.00676127 |
| lvaws | 1 | Echo | Heart | -0.0017749 | -0.2517581 | 0.24820827 | 0.12754477 | 0.02326009 | -0.1776617 | 0.2241819 | 0.10251301 | 0.0112569 | -0.0306073 | 0.0531211 | 0.02135968 |
| lvidd | 1 | Echo | Heart | 0.04532565 | -0.0241892 | 0.11484046 | 0.03546739 | 0.09814501 | 0.0208146 | 0.17547541 | 0.03945501 | 0.05280525 | 0.03786687 | 0.06774363 | 0.00762176 |
| lvids | 1 | Echo | Heart | -0.0635228 | -0.1990947 | 0.07204908 | 0.06917061 | 0.00833519 | -0.1335894 | 0.15025981 | 0.07241185 | 0.07561766 | 0.05257774 | 0.09865758 | 0.01175528 |
| lvpwd | 1 | Echo | Heart | -0.0317376 | -0.1258062 | 0.06233109 | 0.04799509 | -0.0104248 | -0.1271922 | 0.1063426 | 0.05957629 | 0.03026739 | 0.01319002 | 0.04734477 | 0.0087131 |
| lvpws | 1 | Echo | Heart | -0.0190522 | -0.101467 | 0.06336273 | 0.04204919 | 0.00895915 | -0.0823356 | 0.10025395 | 0.04657983 | 0.02684873 | 0.00631461 | 0.04738285 | 0.01047678 |
| magnesium | 1 | Urinalysis | Physiology | 0.01616986 | -0.0231196 | 0.05545927 | 0.02004599 | -0.0513056 | -0.1167021 | 0.0140909 | 0.03336616 | -0.0413354 | -0.113558 | 0.03088709 | 0.03684891 |
| mean cell hemoglobin concentration | 1 | Hematology | Hematology | 0.03780147 | -0.0880637 | 0.16366664 | 0.0642181 | 0.02530629 | -0.1086076 | 0.15922022 | 0.06832469 | -0.011345 | -0.0150702 | -0.0076199 | 0.00190062 |
| mean cell volume | 1 | Hematology | Hematology | 0.00391751 | -0.0957495 | 0.10358452 | 0.05085145 | -0.0030447 | -0.0961742 | 0.09008479 | 0.04751593 | -0.0063502 | -0.0099649 | -0.0027355 | 0.00184428 |
| mean corpuscular hemoglobin | 1 | Hematology | Hematology | -0.0025833 | -0.0653065 | 0.06013979 | 0.03200218 | -0.0193465 | -0.082467 | 0.04377406 | 0.03220494 | -0.0169768 | -0.0197231 | -0.0142305 | 0.0014012 |
| mean platelet volume | 1 | Hematology | Hematology | 0.04873659 | -0.0044688 | 0.10194195 | 0.02714609 | 0.03539134 | -0.0210323 | 0.09181497 | 0.02878809 | -0.0174066 | -0.0276044 | -0.0072089 | 0.00520303 |
| mean r amplitude | 1 | Electrocardiogram (ECG) | Heart | 0.00847034 | -0.0282092 | 0.04514986 | 0.01871438 | -0.0948208 | -0.1630495 | -0.0265922 | 0.03481119 | -0.0835612 | -0.1503108 | -0.0168116 | 0.03405655 |
| mean sr amplitude | 1 | Electrocardiogram (ECG) | Heart | 0.02846175 | -0.0131943 | 0.07011781 | 0.02125348 | -0.0876811 | -0.1270777 | -0.0482845 | 0.02010067 | -0.1130259 | -0.1558048 | -0.070247 | 0.02182636 |
| number of center entries | 1 | Open Field | Behaviour | 0.01507034 | -0.0534907 | 0.08363133 | 0.03498074 | -0.0361259 | -0.0952472 | 0.02299547 | 0.03016451 | -0.0588092 | -0.1679907 | 0.05037225 | 0.05570587 |
| number of rears - total | 1 | Open Field | Behaviour | -0.0011326 | -0.1141113 | 0.11184607 | 0.05764325 | 0.18694902 | -0.0392422 | 0.41314023 | 0.1154058 | 0.17943279 | 0.0568682 | 0.30199739 | 0.0625341 |
| others | 1 | Immunophenotyping | Immunology | -0.1684902 | -0.2596648 | -0.0773156 | 0.0465185 | -0.1515195 | -0.2435956 | -0.0594434 | 0.04697848 | 0.01961583 | 0.00493492 | 0.03429674 | 0.0074904 |
| pdcs | 1 | Immunophenotyping | Immunology | -0.1732553 | -0.4003845 | 0.05387384 | 0.11588436 | -0.2572491 | -0.7186201 | 0.20412192 | 0.23539769 | -0.0915619 | -0.2522236 | 0.06909973 | 0.08197174 |
| percentage center time | 1 | Open Field | Behaviour | -0.0219679 | -0.0863184 | 0.04238259 | 0.03283248 | -0.0188907 | -0.0912088 | 0.0534274 | 0.03689767 | -0.0061802 | -0.0972542 | 0.08489379 | 0.04646717 |
| periphery average speed | 1 | Open Field | Behaviour | -0.0444272 | -0.108287 | 0.0194327 | 0.03258217 | -0.1401304 | -0.2117709 | -0.0684898 | 0.03655197 | -0.0963838 | -0.1446043 | -0.0481633 | 0.02460275 |
| periphery distance travelled | 1 | Open Field | Behaviour | -0.0313217 | -0.0918314 | 0.02918792 | 0.03087284 | -0.1342236 | -0.1874097 | -0.0810376 | 0.02713623 | -0.1037239 | -0.1714836 | -0.0359643 | 0.0345719 |
| periphery permanence time | 1 | Open Field | Behaviour | -0.0369177 | -0.1277076 | 0.05387211 | 0.04632221 | -0.0294978 | -0.1006346 | 0.04163901 | 0.03629496 | 0.00770384 | -0.013785 | 0.02919273 | 0.01096392 |
| periphery resting time | 1 | Open Field | Behaviour | -0.0536346 | -0.1266045 | 0.01933532 | 0.03723022 | -0.0572459 | -0.1071515 | -0.0073404 | 0.02546249 | 0.00260068 | -0.0558538 | 0.06105516 | 0.02982426 |
| phosphorus | 1 | Clinical Chemistry | Physiology | -0.0485897 | -0.0839101 | -0.0132693 | 0.01802092 | -0.082612 | -0.1576473 | -0.0075767 | 0.03828404 | -0.0420616 | -0.0813582 | -0.002765 | 0.02004965 |
| platelet count | 1 | Hematology | Hematology | 0.07371983 | 0.02058624 | 0.12685342 | 0.02710947 | 0.24151349 | 0.18653295 | 0.29649402 | 0.02805181 | 0.16421916 | 0.13698202 | 0.19145629 | 0.01389675 |
| pnn5(6>ms) | 1 | Electrocardiogram (ECG) | Heart | 0.29069047 | 0.17162022 | 0.40976073 | 0.06075125 | -0.2926013 | -0.5272121 | -0.0579905 | 0.11970158 | -0.6004767 | -0.9244113 | -0.276542 | 0.16527583 |
| potassium | 1 | Clinical Chemistry | Physiology | -0.0705522 | -0.2214989 | 0.0803945 | 0.07701505 | -0.0074675 | -0.1729366 | 0.15800151 | 0.08442453 | 0.07041617 | 0.04766471 | 0.09316762 | 0.0116081 |
| pq | 1 | Electrocardiogram (ECG) | Heart | -0.065096 | -0.1538776 | 0.02368566 | 0.04529759 | -0.0648322 | -0.1270688 | -0.0025955 | 0.03175399 | 0.00156562 | -0.0259865 | 0.02911777 | 0.01405748 |
| pr | 1 | Electrocardiogram (ECG) | Heart | -0.056486 | -0.1048371 | -0.0081349 | 0.02466939 | -0.0754718 | -0.1235224 | -0.0274213 | 0.02451604 | -0.0183785 | -0.0319887 | -0.0047684 | 0.00694408 |
| qrs | 1 | Electrocardiogram (ECG) | Heart | 0.07254538 | 0.03547224 | 0.10961853 | 0.01891522 | 0.06810735 | 0.03008685 | 0.10612785 | 0.01939857 | -0.0054233 | -0.0154885 | 0.00464183 | 0.00513537 |
| qtc | 1 | Electrocardiogram (ECG) | Heart | 0.03281061 | -0.0101032 | 0.07572442 | 0.0218952 | 0.03104729 | -0.0207365 | 0.08283105 | 0.02642077 | -5.05E-04 | -0.0085696 | 0.00756037 | 0.00411485 |
| qtc dispersion | 1 | Electrocardiogram (ECG) | Heart | 0.00312581 | -0.0523919 | 0.05864351 | 0.02832588 | -0.0046501 | -0.106053 | 0.09675281 | 0.05173714 | -0.0077373 | -0.0510162 | 0.03554163 | 0.02208148 |
| red blood cell count | 1 | Hematology | Hematology | 0.0773455 | 0.0071933 | 0.1474977 | 0.0357926 | 0.09972781 | 0.03169959 | 0.16775602 | 0.03470891 | 0.02284931 | 0.00885827 | 0.03684035 | 0.00713842 |
| red blood cell distribution width | 1 | Hematology | Hematology | 0.12484642 | -0.0035148 | 0.25320764 | 0.06549162 | 0.135346 | -0.0035862 | 0.27427818 | 0.07088507 | 0.01047894 | -0.0032056 | 0.02416352 | 0.00698206 |
| respiration rate | 1 | Echo | Heart | -0.1384843 | -0.2178736 | -0.059095 | 0.04050548 | -0.070357 | -0.1795875 | 0.03887346 | 0.05573086 | 0.06110337 | 0.02271414 | 0.09949259 | 0.0195867 |
| respiratory exchange ratio | 1 | Indirect Calorimetry | Metabolism | -0.0116565 | -0.089649 | 0.06633605 | 0.03979283 | -0.010653 | -0.0878483 | 0.06654237 | 0.0393861 | 0.00170269 | -0.0057348 | 0.00914021 | 0.00379472 |
| right anterior chamber depth | 1 | Eye Morphology | Eye | -0.4491432 | -1.3293546 | 0.43106822 | 0.44909572 | -0.4157377 | -1.291862 | 0.46038667 | 0.44701043 | 0.03160985 | 0.02645119 | 0.0367685 | 0.00263202 |
| right corneal thickness | 1 | Eye Morphology | Eye | -0.0355898 | -0.2280522 | 0.1568726 | 0.09819692 | -0.030655 | -0.1963692 | 0.13505925 | 0.08454964 | -0.0013855 | -0.023783 | 0.02101208 | 0.01142753 |
| right inner nuclear layer | 1 | Eye Morphology | Eye | -0.2545083 | -0.7633116 | 0.25429494 | 0.25959828 | -0.2785114 | -0.8373133 | 0.28029065 | 0.2851083 | -0.017509 | -0.0664158 | 0.03139785 | 0.02495292 |
| right outer nuclear layer | 1 | Eye Morphology | Eye | 0.00612525 | -0.0781241 | 0.0903746 | 0.04298515 | 0.01090977 | -0.0731427 | 0.09496221 | 0.04288468 | 0.00555134 | 5.19E-05 | 0.01105079 | 0.00280589 |
| right posterior chamber depth | 1 | Eye Morphology | Eye | -0.0775673 | -0.2905688 | 0.13543413 | 0.10867622 | -0.0764571 | -0.2893152 | 0.13640097 | 0.10860305 | 0.00719898 | -0.0178434 | 0.03224132 | 0.01277694 |
| right total retinal thickness | 1 | Eye Morphology | Eye | -0.1987993 | -0.645732 | 0.24813332 | 0.22803105 | -0.1925482 | -0.6285715 | 0.24347499 | 0.22246492 | 0.00528817 | -0.0045957 | 0.01517202 | 0.00504287 |
| rmssd | 1 | Electrocardiogram (ECG) | Heart | 0.18002732 | -0.0882317 | 0.44828637 | 0.13686938 | -0.0161048 | -0.4112809 | 0.3790712 | 0.20162414 | -0.1178703 | -0.2449843 | 0.00924365 | 0.06485525 |
| rp macrophage (cd19- cd11c-) | 1 | Immunophenotyping | Immunology | -0.0765771 | -0.3398075 | 0.18665328 | 0.13430368 | -0.0747691 | -0.3351316 | 0.18559334 | 0.13284044 | -0.0746396 | -0.207298 | 0.05801883 | 0.0676841 |
| rr | 1 | Electrocardiogram (ECG) | Heart | -0.0761505 | -0.1876687 | 0.03536782 | 0.05689812 | -0.0896869 | -0.2063458 | 0.02697206 | 0.05952096 | -0.0125023 | -0.0214082 | -0.0035963 | 0.00454396 |
| sodium | 1 | Clinical Chemistry | Physiology | 0.02620997 | -0.1171674 | 0.16958732 | 0.07315305 | 0.0338228 | -0.1337162 | 0.20136179 | 0.08548065 | 0.00996804 | 0.00658153 | 0.01335454 | 0.00172784 |
| spleen weight | 1 | Immunophenotyping | Immunology | 0.18742593 | -0.0500875 | 0.42493933 | 0.12118254 | 0.11337064 | -0.1604807 | 0.38722201 | 0.13972265 | -0.1542349 | -0.2104415 | -0.0980283 | 0.02867736 |
| st | 1 | Electrocardiogram (ECG) | Heart | 0.00328878 | -0.0544512 | 0.06102879 | 0.02945973 | -0.0054976 | -0.081181 | 0.0701858 | 0.03861468 | -0.0034902 | -0.0175917 | 0.01061128 | 0.00719476 |
| stroke volume | 1 | Echo | Heart | 0.05942758 | -0.0782445 | 0.1970997 | 0.07024217 | 0.157433 | 0.00918914 | 0.30567685 | 0.07563601 | 0.09373745 | 0.07755874 | 0.10991617 | 0.0082546 |
| tibia length | 1 | Heart Weight | Morphology | -0.1475403 | -0.4396127 | 0.144532 | 0.14901924 | -0.1374401 | -0.4261352 | 0.15125505 | 0.14729614 | 0.00951991 | 0.00591986 | 0.01311997 | 0.00183679 |
| total bilirubin | 1 | Clinical Chemistry | Physiology | 0.06054492 | -0.0097669 | 0.13085674 | 0.03587403 | 0.00226712 | -0.085991 | 0.09052519 | 0.04503046 | -0.0550333 | -0.0979518 | -0.0121148 | 0.0218976 |
| total cholesterol | 1 | Clinical Chemistry | Physiology | 0.09425949 | -0.0751596 | 0.26367863 | 0.08643992 | 0.31422081 | 0.11256129 | 0.51588034 | 0.1028894 | 0.20275827 | 0.1750477 | 0.23046884 | 0.01413831 |
| total food intake | 1 | Indirect Calorimetry | Metabolism | -0.1192293 | -0.2542902 | 0.01583164 | 0.06890989 | -0.0964842 | -0.2564912 | 0.06352279 | 0.08163771 | 0.02676913 | -0.0233285 | 0.07686674 | 0.02556048 |
| total protein | 1 | Clinical Chemistry | Physiology | -0.0422347 | -0.0623878 | -0.0220816 | 0.01028239 | -0.0355909 | -0.0619127 | -0.0092692 | 0.01342971 | 0.00926598 | -8.16E-04 | 0.01934777 | 0.00514386 |
| total water intake | 1 | Indirect Calorimetry | Metabolism | -0.1457383 | -0.2373165 | -0.0541601 | 0.04672441 | -0.2097443 | -0.2681948 | -0.1512937 | 0.02982226 | -0.0654284 | -0.137422 | 0.00656531 | 0.03673213 |
| triglycerides | 1 | Clinical Chemistry | Physiology | -0.032002 | -0.1233659 | 0.05936187 | 0.04661509 | 0.3268957 | 0.2087111 | 0.4450803 | 0.06029937 | 0.34735523 | 0.25920061 | 0.43550985 | 0.04497767 |
| urea (blood urea nitrogen - bun) | 1 | Clinical Chemistry | Physiology | -0.1405306 | -0.266412 | -0.0146491 | 0.06422641 | -0.095004 | -0.2507897 | 0.0607817 | 0.07948396 | 0.0403162 | 0.00518826 | 0.07544413 | 0.01792274 |
| uric acid | 1 | Clinical Chemistry | Physiology | 0.03670624 | -0.0660619 | 0.13947443 | 0.05243371 | 0.36269571 | 0.09145118 | 0.63394024 | 0.13839261 | 0.44723487 | -0.0801891 | 0.97465879 | 0.26909878 |
| white blood cell count | 1 | Hematology | Hematology | -0.0907957 | -0.1703063 | -0.0112852 | 0.04056734 | 0.11684459 | -0.0023934 | 0.23608259 | 0.06083683 | 0.19788757 | 0.13683048 | 0.25894465 | 0.03115215 |
| whole arena average speed | 1 | Open Field | Behaviour | -0.0156634 | -0.0857564 | 0.05442959 | 0.0357624 | -0.1140149 | -0.1840029 | -0.0440269 | 0.03570882 | -0.0997437 | -0.1519566 | -0.0475307 | 0.02663974 |
| whole arena resting time | 1 | Open Field | Behaviour | -0.0531307 | -0.1011672 | -0.0050941 | 0.0245089 | -0.0593672 | -0.1076067 | -0.0111276 | 0.02461247 | 0.00458779 | -0.0513396 | 0.0605152 | 0.02853492 |

Table 4: Summary of overall meta-analyses on the functional trait group level (GroupingTerm). Results for lnCVR, lnVR and lnRR and their respective upper and lower 95 percent CI’s, standard error and I2 values are provided. Values truncated at 5 decimal places for readability.

| Grouping  Term | lnCVR | lnCVR_  lower | lnCVR_  upper | lnCVR_se | lnCVR_I2 | lnVR | lnVR_  lower | lnVR_  upper | lnVR_se | lnVR_I2 | lnRR | lnRR_  lower | lnRR_  upper | lnRR_se | lnRR_I2 |
| --- | --- | --- | --- | --- | --- | --- | --- | --- | --- | --- | --- | --- | --- | --- | --- |
| Behaviour | -0.00350 | -0.02407 | 0.01706 | 0.01049 | 39.03280 | -0.01783 | -0.07399 | 0.03832 | 0.02865 | 92.79916 | -0.01992 | -0.06344 | 0.02360 | 0.02220 | 89.23815 |
| Morphology | 0.07745 | 0.04142 | 0.11347 | 0.01838 | 67.28181 | 0.15142 | 0.08188 | 0.22095 | 0.03548 | 90.62639 | 0.06782 | 0.00722 | 0.12841 | 0.03092 | 99.69834 |
| Metabolism | -0.04308 | -0.11259 | 0.02643 | 0.03547 | 84.80072 | 0.09106 | -0.03377 | 0.21589 | 0.06369 | 96.78300 | 0.14226 | 0.03644 | 0.24808 | 0.05399 | 99.37741 |
| Physiology | 0.01268 | -0.01401 | 0.03937 | 0.01362 | 70.56587 | 0.03598 | -0.02779 | 0.09976 | 0.03254 | 90.64322 | 0.01637 | -0.04434 | 0.07708 | 0.03097 | 99.81718 |
| Immunology | -0.06818 | -0.09801 | -0.03835 | 0.01522 | 40.76943 | -0.11124 | -0.16221 | -0.06026 | 0.02601 | 58.01960 | -0.05748 | -0.10742 | -0.00755 | 0.02548 | 96.14795 |
| Hematology | 0.02179 | -0.01650 | 0.06008 | 0.01954 | 60.75258 | 0.08021 | 0.03154 | 0.12888 | 0.02483 | 64.94307 | 0.03885 | -0.00243 | 0.08013 | 0.02106 | 99.63011 |
| Heart | 0.01838 | -0.01284 | 0.04961 | 0.01593 | 81.29798 | -0.00508 | -0.03570 | 0.02554 | 0.01562 | 74.05785 | -0.00489 | -0.03242 | 0.02264 | 0.01405 | 98.98516 |
| Hearing | 0.01573 | -0.01120 | 0.04266 | 0.01374 | 21.75599 | 0.01069 | -0.02304 | 0.04442 | 0.01721 | 22.79692 | -0.01324 | -0.03360 | 0.00713 | 0.01039 | 79.48601 |
| Eye | -0.08179 | -0.14768 | -0.01590 | 0.03362 | 49.80988 | -0.07445 | -0.13814 | -0.01076 | 0.03249 | 48.30933 | 0.00912 | 0.00121 | 0.01703 | 0.00404 | 90.52849 |
| All | 0.00466 | -0.00862 | 0.01793 | 0.00678 | 76.48552 | 0.01566 | -0.00775 | 0.03907 | 0.01194 | 90.75958 | 0.01243 | -0.00615 | 0.03101 | 0.00948 | 99.71605 |

Table 5: Provides an overview of meta-analysis results performed on traits that were significantly biased towards either sex. This table summarizes findings for both sexes and the respective functional trait groups. Values truncated at 5 decimal places for readability.

| Grouping Term | Sex | lnCVR | lnCVR_ lower | lnCVR_ upper | lnCVR_ se | lnCVR_ I2 | lnVR | lnVR_ lower | lnVR_ upper | lnVR_ se | lnVR_ I2 | lnRR | lnRR_ lower | lnRR_u pper | lnRR_se | lnRR_I2 |
| --- | --- | --- | --- | --- | --- | --- | --- | --- | --- | --- | --- | --- | --- | --- | --- | --- |
| Behaviour | female | -0.05313 | -0.10117 | -0.00509 | 0.02451 | 0.00000 | -0.09300 | -0.12048 | -0.06551 | 0.01402 | 42.13880 | -0.09447 | -0.11678 | -0.07216 | 0.01138 | 0.00000 |
| Behaviour | male | 0.10743 | 0.04986 | 0.16499 | 0.02937 | 0.00000 | 0.28357 | 0.18943 | 0.37772 | 0.04804 | 37.80779 | 0.20315 | 0.13463 | 0.27167 | 0.03496 | 0.00000 |
| Morphology | female | NA | NA | NA | NA | NA | NA | NA | NA | NA | NA | -0.10963 | -0.22983 | 0.01056 | 0.06133 | 93.93533 |
| Morphology | male | 0.12709 | 0.08622 | 0.16796 | 0.02085 | 41.54558 | 0.21740 | 0.15488 | 0.27992 | 0.03190 | 84.37777 | 0.12039 | 0.05789 | 0.18288 | 0.03189 | 99.67361 |
| Metabolism | female | -0.12453 | -0.16624 | -0.08282 | 0.02128 | 13.32401 | -0.20974 | -0.26819 | -0.15129 | 0.02982 | 0.00000 | NA | NA | NA | NA | NA |
| Metabolism | male | 0.09391 | 0.05200 | 0.13583 | 0.02139 | 0.00000 | 0.22421 | 0.10018 | 0.34825 | 0.06328 | 95.76071 | 0.22261 | 0.10986 | 0.33537 | 0.05753 | 98.64730 |
| Physiology | female | -0.04718 | -0.06401 | -0.03034 | 0.00859 | 0.61017 | -0.16326 | -0.29629 | -0.03023 | 0.06787 | 92.84326 | -0.11710 | -0.22070 | -0.01351 | 0.05286 | 99.30119 |
| Physiology | male | 0.09638 | 0.06304 | 0.12972 | 0.01701 | 0.10939 | 0.23959 | 0.15950 | 0.31968 | 0.04086 | 56.00632 | 0.14458 | 0.07081 | 0.21836 | 0.03764 | 98.51821 |
| Immunology | female | -0.09005 | -0.14809 | -0.03201 | 0.02961 | 81.54813 | -0.17975 | -0.21812 | -0.14138 | 0.01958 | 0.00000 | -0.15926 | -0.22860 | -0.08992 | 0.03538 | 94.72909 |
| Immunology | male | NA | NA | NA | NA | NA | NA | NA | NA | NA | NA | 0.02825 | 0.00133 | 0.05517 | 0.01373 | 44.58750 |
| Hematology | female | -0.09204 | -0.15019 | -0.03389 | 0.02967 | 0.00000 | NA | NA | NA | NA | NA | -0.01251 | -0.01783 | -0.00718 | 0.00272 | 84.99027 |
| Hematology | male | 0.08244 | 0.04820 | 0.11667 | 0.01747 | 0.08911 | 0.14443 | 0.04558 | 0.24328 | 0.05043 | 86.35043 | 0.11586 | 0.02655 | 0.20517 | 0.04557 | 98.44500 |
| Heart | female | -0.09119 | -0.17060 | -0.01179 | 0.04051 | 66.54844 | -0.08391 | -0.10927 | -0.05855 | 0.01294 | 0.52801 | -0.10762 | -0.17889 | -0.03635 | 0.03636 | 98.81450 |
| Heart | male | 0.15417 | 0.08512 | 0.22322 | 0.03523 | 78.69922 | 0.10406 | 0.06191 | 0.14620 | 0.02150 | 36.41233 | 0.05785 | 0.04114 | 0.07456 | 0.00853 | 84.64962 |
| Hearing | female | NA | NA | NA | NA | NA | NA | NA | NA | NA | NA | -0.03543 | -0.06219 | -0.00868 | 0.01365 | 61.36216 |
| Hearing | male | NA | NA | NA | NA | NA | 0.08696 | 0.00658 | 0.16735 | 0.04101 | 0.00000 | 0.01840 | 0.00569 | 0.03112 | 0.00649 | 0.00000 |
| Eye | female | -0.16402 | -0.24986 | -0.07818 | 0.04379 | 3.07113 | -0.16646 | -0.27724 | -0.05568 | 0.05652 | 24.07626 | NA | NA | NA | NA | NA |
| Eye | male | NA | NA | NA | NA | NA | NA | NA | NA | NA | NA | 0.01921 | 0.00424 | 0.03418 | 0.00764 | 97.04593 |
| All | female | -0.08669 | -0.10925 | -0.06414 | 0.01151 | 66.47942 | -0.13025 | -0.15733 | -0.10318 | 0.01382 | 75.91923 | -0.09849 | -0.12891 | -0.06807 | 0.01552 | 99.61166 |
| All | male | 0.11134 | 0.09309 | 0.12960 | 0.00931 | 34.06982 | 0.19755 | 0.16183 | 0.23326 | 0.01822 | 86.49690 | 0.11088 | 0.08327 | 0.13850 | 0.01409 | 99.63948 |

Table 6: Summarizes our findings on heterogeneity due to institutions and mouse strains. These results are based on meta-analyses on sigmaˆ2 and errors for mouse strains and centers (Institutions), following the identical workflow from above. Values truncated at 5 decimal places for readability.

| Grouping Term | lnCVR | lnCVR_ lower | lnCVR_ higher | lnVR | lnVR_ lower | lnVR_ higher | lnRR | lnRR_ lower | lnRR_ higher | CVR | CVR_l ower | CVR_ higher | VR | VR_ l ower | VR_ h igher | RR | RR_ lower | RR_ higher |
| --- | --- | --- | --- | --- | --- | --- | --- | --- | --- | --- | --- | --- | --- | --- | --- | --- | --- | --- |
| Behaviour | -1.64913 | -2.46361 | -0.83465 | -1.54485 | -2.35933 | -0.73037 | -2.64039 | -4.13538 | -1.14540 | 0.19222 | 0.08513 | 0.43403 | 0.21334 | 0.09448 | 0.48173 | 0.07133 | 0.01600 | 0.31810 |
| Morpho-logy | -1.74512 | -2.42423 | -1.06601 | -1.72516 | -2.40426 | -1.04605 | -7.27698 | -10.00450 | -4.54947 | 0.17462 | 0.08855 | 0.34438 | 0.17815 | 0.09033 | 0.35132 | 0.00069 | 0.00005 | 0.01057 |
| Meta-bolism | -2.40352 | -3.56742 | -1.23961 | -2.89723 | -4.26003 | -1.53444 | -2.97039 | -4.13429 | -1.80649 | 0.09040 | 0.02823 | 0.28950 | 0.05518 | 0.01412 | 0.21558 | 0.05128 | 0.01601 | 0.16423 |
| Physiology | -2.89932 | -3.76207 | -2.03657 | -1.92151 | -2.68774 | -1.15527 | -2.66380 | -3.43004 | -1.89756 | 0.05506 | 0.02324 | 0.13048 | 0.14639 | 0.06803 | 0.31497 | 0.06968 | 0.03239 | 0.14993 |
| Immuno-logy | -0.29073 | -0.79474 | 0.21329 | -0.23132 | -0.73533 | 0.27269 | -0.89472 | -1.79007 | 0.00064 | 0.74772 | 0.45170 | 1.23774 | 0.79349 | 0.47935 | 1.31350 | 0.40872 | 0.16695 | 1.00064 |
| Hemato-logy | -0.46303 | -1.11953 | 0.19347 | -0.36902 | -1.02552 | 0.28748 | -1.28687 | -2.21998 | -0.35377 | 0.62937 | 0.32643 | 1.21345 | 0.69141 | 0.35861 | 1.33307 | 0.27613 | 0.10861 | 0.70204 |
| Heart | -3.57289 | -4.27204 | -2.87374 | -2.97974 | -3.54853 | -2.41095 | -3.87694 | -4.44336 | -3.31051 | 0.02807 | 0.01395 | 0.05649 | 0.05081 | 0.02877 | 0.08973 | 0.02071 | 0.01176 | 0.03650 |
| Hearing | -3.05850 | -5.04933 | -1.06767 | -2.36128 | -4.35211 | -0.37044 | -3.60160 | -5.59244 | -1.61077 | 0.04696 | 0.00641 | 0.34381 | 0.09430 | 0.01288 | 0.69043 | 0.02728 | 0.00373 | 0.19973 |
| Eye | -3.70685 | -5.10166 | -2.31205 | -3.22781 | -4.33106 | -2.12456 | -5.77287 | -6.57400 | -4.97175 | 0.02455 | 0.00609 | 0.09906 | 0.03964 | 0.01315 | 0.11949 | 0.00311 | 0.00140 | 0.00693 |
| All | -2.28981 | -2.64805 | -1.93157 | -1.93647 | -2.25407 | -1.61887 | -3.45233 | -3.94443 | -2.96022 | 0.10129 | 0.07079 | 0.14492 | 0.14421 | 0.10497 | 0.19812 | 0.03167 | 0.01936 | 0.05181 |
