# Supplementary material for: Sexual dimorphism in trait variability and its eco-evolutionary and statistical implications
Source: eLife. 2020 Nov 17;9:e63170. doi: 10.7554/eLife.63170 (PMC7704105; doi:10.7554/eLife.63170)
Supplement: Transparent reporting form [file elife-63170-transrepform.docx]

***eLife’s* transparent reporting form**

We encourage authors to provide detailed information *within their submission* to facilitate the interpretation and replication of experiments. Authors can upload supporting documentation to indicate the use of appropriate reporting guidelines for health-related research (see [EQUATOR Network](http://www.equator-network.org/%20)), life science research (see the [BioSharing Information Resource](https://biosharing.org/)), or the [ARRIVE guidelines](http://www.plosbiology.org/article/info:doi/10.1371/journal.pbio.1000412) for reporting work involving animal research. Where applicable, authors should refer to any relevant reporting standards documents in this form.

If you have any questions, please consult our Journal Policies and/or contact us: [editorial@elifesciences.org](mailto:editorial@elifesciences.org).

**Sample-size estimation**

- You should state whether an appropriate sample size was computed when the study was being designed
- You should state the statistical method of sample size computation and any required assumptions
- If no explicit power analysis was used, you should describe how you decided what sample (replicate) size (number) to use

Our manuscript is based on data generated by the IMPC. Our aim was to investigate variability in traits in a sex-specific manner. The cleaned-up data set contains information on 27 000 mice. As part of our submission, we provide a shinyApp that may help researchers adjust for sex-specific bias in certain procedures and traits in their calculations for optimal sample sizes.

Please outline where this information can be found within the submission (e.g., sections or figure legends), or explain why this information doesn’t apply to your submission:

**Replicates**

- You should report how often each experiment was performed
- You should include a definition of biological versus technical replication
- The data obtained should be provided and sufficient information should be provided to indicate the number of independent biological and/or technical replicates
- If you encountered any outliers, you should describe how these were handled
- Criteria for exclusion/inclusion of data should be clearly stated
- High-throughput sequence data should be uploaded before submission, with a private link for reviewers provided (these are available from both GEO and ArrayExpress)

Please outline where this information can be found within the submission (e.g., sections or figure legends), or explain why this information doesn’t apply to your submission:

As this work is based on existing data this doesn’t apply.

We define mice that were investigated in different institutions as different “populations”. Details can be found in the first two paragraphs of the Methods section, as well as part “2.2.3 Population statistics” of the supporting html file (<https://rpubs.com/SusZaj/ESF>); which together with our fully annotated code clearly outlines all procedures: 10.5281/zenodo.4146948 . Openly accessible code and resource files can be also accessed via OSF <https://osf.io/25h4t/> and GitHub <https://github.com/itchyshin/mice_sex_diff>

**Statistical reporting**

- Statistical analysis methods should be described and justified
- Raw data should be presented in figures whenever informative to do so (typically when N per group is less than 10)
- For each experiment, you should identify the statistical tests used, exact values of N, definitions of center, methods of multiple test correction, and dispersion and precision measures (e.g., mean, median, SD, SEM, confidence intervals; and, for the major substantive results, a measure of effect size (e.g., Pearson's r, Cohen's d)
- Report exact p-values wherever possible alongside the summary statistics and 95% confidence intervals. These should be reported for all key questions and not only when the p-value is less than 0.05.

Please outline where this information can be found within the submission (e.g., sections or figure legends), or explain why this information doesn’t apply to your submission:

We use meta-analytical methods to investigate differences in male and female responses to a wide range of traits and procedures. The “Methods” section clearly outlines how we calculate effect sizes. We provide a fully reproducible R script on <https://rpubs.com/SusZaj/ESF> , <https://github.com/itchyshin/mice_sex_diff> and <https://osf.io/25h4t/>

(For large datasets, or papers with a very large number of statistical tests, you may upload a single table file with tests, Ns, etc., with reference to sections in the manuscript.)

**Group allocation**

- Indicate how samples were allocated into experimental groups (in the case of clinical studies, please specify allocation to treatment method); if randomization was used, please also state if restricted randomization was applied
- Indicate if masking was used during group allocation, data collection and/or data analysis

Not applicable. We use data from the IMPC, pre-selected only for non-binary traits and available for both sexes.

Please outline where this information can be found within the submission (e.g., sections or figure legends), or explain why this information doesn’t apply to your submission:

**Additional data files (“source data”)**

- We encourage you to upload relevant additional data files, such as numerical data that are represented as a graph in a figure, or as a summary table
- Where provided, these should be in the most useful format, and they can be uploaded as “Source data” files linked to a main figure or table
- Include model definition files including the full list of parameters used
- Include code used for data analysis (e.g., R, MatLab)
- Avoid stating that data files are “available upon request”

The entire workflow, R script and necessary data are provided: All data and code are freely accessible: code, associated files, processed data: <https://osf.io/25h4t/>, https://github.com/itchyshin/mice_sex_diff https://rpubs.com/SusZaj/ESF

Raw data (large file size): <https://doi.org/10.5281/zenodo.3759701>

Our workflow builds up on previous steps in the script, making it a little difficult to provide separate files for all figures. We have provided the source data for Figure 4 (source file 1 for 4A, source file 2 is associated with 4B).

Please indicate the figures or tables for which source data files have been provided:
